# Effect of Terbuthylazine-2-hydroxy at Environmental Concentrations on Early Life Stages of Common Carp (*Cyprinus carpio* L.)

**DOI:** 10.1155/2014/621304

**Published:** 2014-02-06

**Authors:** Josef Velisek, Alzbeta Stara, Dalibor Koutnik, Jana Machova

**Affiliations:** Research Institute of Fish Culture and Hydrobiology, South Bohemian Research Center of Aquaculture and Biodiversity of Hydrocenoses, Faculty of Fisheries and Protection of Waters, University of South Bohemia in Ceske Budejovice, Zatisi 728/II, 389 25 Vodnany, Czech Republic

## Abstract

The aim of the study was to investigate effects of the triazine's herbicide terbuthylazine-2-hydroxy on early life stage of common carp (*Cyprinus carpio* L.) through antioxidant indices, mortality, growth, development, and histopathology. Based on accumulated mortality in the experimental groups, lethal concentrations of terbuthylazine-2-hydroxy were estimated at 35-day LC50 = 10.9 mg/L terbuthylazine-2-hydroxy. By day 15, fish were exposed to 3.5 mg/L and by day 26, fish were exposed to 0.0029 mg/L; real environmental concentration in Czech rivers, 0.07 mg/L, 1.4 mg/L, and 3.5 mg/L terbuthylazine-2-hydroxy, showed significantly lower mass and total length compared with controls. Based on inhibition of growth in the experimental groups, lowest observed effect concentration (LOEC) = 0.002 mg/L terbuthylazine-2-hydroxy and no observed effect concentration (NOEC) = 0.0001 mg/L terbuthylazine-2-hydroxy. No significant negative effects on hatching or embryo viability were demonstrated at the concentrations tested, but significant differences in early ontogeny among groups were noted. Fish from the two highest tested concentrations showed a dose-related delay in development compared with the controls. Total superoxide dismutase (SOD) activity was significant lower in all groups testedly for terbuthylazine-2-hydroxy compared with the control group. At concentrations of 1.4 and 3.5 mg/L damage to caudal kidney tubules when compared to control fish was found.

## 1. Introduction

Triazines are selective herbicides applied before and after emergence control against annual weeds, perennial weeds, grasses, and broadleaf weeds in corn, wheat, sorghum, and many other crops. Terbuthylazine was registered in the United States in 1975 and now is the second most frequently used s-triazine. Due to the persistency, water solubility, and mobility, triazines are also detected in aquatic ecosystems. These compounds are found in rivers, lakes, and well water [1–3]. In water terbutryne and terbuthylazine undergo a variety of biotic and abiotic mechanisms of degradation such as photodegradation, oxidation, hydrolysis, and biodegradation that lead to dealkylation of alkylated amino groups, deamination, and hydroxylation at the 2 position, as well as triazine ring cleavage [4–6]. The major degradation products in ground and surface waters are the terbuthylazine-2-hydroxy, terbuthylazine-desethyl-hydroxy, and terbuthylazine-desethyl. The persistence of these metabolites ranged from moderate to high for terbuthylazine-desethyl-hydroxy, terbuthylazine-desethyl and high to very high for terbuthylazine-2-hydroxy (112–120 days) [7].

Currently seven s-triazines have been identified as relevant in a study of the prioritization of substances dangerous to the aquatic environment in the member states of the European community and are included in the EU Priority Pollutants List and the US Environmental Protection Agency's List. According to Commission Regulation (EU) number 196/2010 of 9 March 2010, amending Annex I to Regulation (EC) number 689/2008 of the European Parliament and of the Council concerning the export and import of dangerous chemicals is banned in the countries of the European Union. Although the effects of atrazine, another s-triazine herbicide exposure to fish, have been well-documented, there is a dearth of data of their metabolites in early life stages of common carp. The aim of the present study was to describe lethal and sublethal effects of terbuthylazine-2-hydroxy on embryo and larvae of common carp using a 35-day embryo larval toxicity test. Toxicity tests with early life stages of aquatic organisms have been proposed as a faster and more cost-efficient way of testing chemicals and environmental samples. Moreover, experience shows that these developmental stages of fishes are often the most sensitive to toxic effects, although the various embryonic and larval stages differ in their susceptibility due to of physiological and biochemical differences. Newly hatched larvae constitute a particularly critical and sensitive life stage, since at hatching the embryos lose their protective membrane and are fully exposed to potential toxicants [8–11]. The toxicity of terbuthylazine-2-hydroxy was assessed on the basis of mortality, early ontogeny, occurrence of morphological anomalies, growth rate, Fulton's weight condition factor (FWC), and the antioxidant defenses during and at the conclusion of the test. The aim of the present study was to investigate how low concentrations can affect terbuthylazine-2-hydroxy on early life stages of carp after long-term exposure.

## 2. Materials and Methods

### 2.1. Experimental Animals

Fertilized eggs of common carp (*Cyprinus carpio* L.) were obtained from the breeding station of the Research Institute of Fish Culture and Hydrobiology in Vodnany, University of South Bohemia (Czech Republic). Eggs were produced according to standard methods of artificial reproduction by mating 15 females with 25 males (full-factorial scheme of crossing) as described by Kocour et al. [12]. Sampling was done randomly from a homogenized batch of eggs, so possible genetic and/or maternal effects on the results of the trial were minimized.

### 2.2. Water Parameters

Aerated tap water was used, with the following parameters: dissolved oxygen > 85%, temperature 19.0–22.0°C, pH 7.6–8.1, ANC_4.5_ (acid neutralization capacity) 0.92 mmol/L, COD_Mn_ (chemical oxygen demand) 0.6 mg/L, total ammonia 0.02 mg/L, NO_3_^−^ 1.50 mg/L, NO_2_^−^ 0.05 mg/L, and sum of Ca^2+^ + Mg^2+^ 4.2 mg/L. The test baths were gently aerated on a continual basis. Oxygen saturation, pH, and temperature were measured daily. Terbuthylazine-2-hydroxy concentrations were checked daily by high performance liquid chromatography (HPLC). The water was chromatographed on a reverse phase HPLC column (Lichrosphere 100 RP_18_, Vertex column, pore size 100 *μ*m, particle size 5 *μ*m, and 250 mm × 5 mm ID) using a solvent system of methanol : water : ammonium acetate 70 : 30 : 0.2 and 80 : 20 : 0.2 (v : v : v) isocratically, at a flow rate of 0.7 mL min⁡^−1^. Injection volumes of samples were 100 *μ*L per injection. UV detection was recorded at 230 nm. Column eluents (1 min fractions) were collected in scintillation vials using a fraction collector (LKB 2212 HeliRac; Amersham Pharmacia Biotech, Freiburg), dissolved in a scintillation cocktail, and counted by LSC. Water samples were assayed using the method of Richter and Nagel [13]. Measured values did not differ from the value stated for test purposes by more than 10%.

### 2.3. Experimental Protocol

The trial was carried out using the modified test design of the Organization for Economic Cooperation and Development Guidelines for Testing of Chemicals 210. At 24 h after fertilization, unfertilized eggs were discarded, and 100 eggs were randomly transferred into fifteen crystallization basins containing the test solutions of terbuthylazine-2-hydroxy (Sigma Aldrich, Czech Republic, chemical purity 99.5%) as well as into a control dish. Four ascending concentrations of test solutions and a control were used, each with 100 fertilized eggs in triplicate groups. The concentrations were as follows: 0.0029 mg/L (reported environmental concentration in Czech rivers, Group 1-E1), 0.07 mg/L (Group 2-E2), 1.4 mg/L (Group 3-E3), and 3.5 mg/L (Group 4-E4). Terbuthylazine-2-hydroxy concentrations of 0.07 mg/L, 1.4 mg/L, and 3.5 mg/L corresponded to the 1% 96hLC50, 20% 96hLC50, and 50% 96hLC50 for carp.

The basins were placed in a laboratory (open-air conditions) with the natural light exposure (16 : 8 h light : dark). The arrangement of basins was random. The water for each treatment was renewed twice daily by gentle draining each chamber and adding new solution slowly to prevent disturbing embryos and larvae. Observations of hatching, survival, and behavior were made twice daily and dead embryos and larvae were removed. When able to feed, larvae were given freshly hatched, tap-water-rinsed brine shrimp (*Artemia salina*) nauplii *ad libitum* twice daily prior to water exchange. The nauplii were rinsed with tap water to avoid contaminating the exposure water with chloride.

During and at the conclusion of the trial samples of embryos and larvae were collected to monitor development, occurrence of morphological anomalies, rate of length and weight increase, FWC, and the length/weight relationship. Samples were collected on days 9, 15, 22, 26, 33, and 35. Samples were fixed in 4% formalin, with 5 specimens per replicate (i.e., 15 per group).

Determination of developmental periods and stages followed Penaz et al. [14], who described nine embryonic (E1-E9), six larval (L1–L6), and two juvenile stages (J1-J2). Final measurements included accumulated mortality, basic length parameters for fish with no cranial/skeletal deformities (TL, total length; SL, standard length), and mass (W).

The length parameters were measured under a stereomicroscope (Olympus SZ61/SZ51) using a micrometer (accuracy of 0.01 mm). Weight to 0.1 mg was measured by using a Mettler-Toledo balance.

### 2.4. Trial Schedule

The trial schedule was as follows: day 1, trial beginning (1 day after fertilization of eggs); day 6, hatching completed; day 9, beginning of exogenous feeding (*A. salina*); day 35, end of the trial (at that time, the majority of fish in the control group had reached the first juvenile stage).

### 2.5. Growth Rate Evaluation

The mean specific growth rate (SGR) for fish in each of the experimental groups was calculated for the period beginning on day 9 (the first sampling time) and ending on day 35 (end of the trial). The following formula was used:

(1) $$\begin{array}{c} \text{SGR}=\frac{\bar{\ln w_{2}}-\bar{\ln w_{1}}}{t_{2}-t_{1}}\cdot 100, \end{array}$$

where SGR = mean specific growth rate in the group, *w*_1_ = mass of one fish at time *t*_1_ individually (*μ*g), *w*_2_ = mass of one fish at time *t*_2_ individually (*μ*g), $\bar{\ln w_{1}}$ = mean value of the ln⁡*w*_1_ values, $\bar{\ln w_{2}}$ = mean value of the ln⁡*w*_2_ values, *t*_1_ = time (days)-first sampling time, and *t*_2_ = time (days)-end of exposure.

The inhibition of specific growth rate in each experimental group was calculated as follows:

(2) $$\begin{array}{c} I_{x}\left[\%\right]=\frac{\text{SG}{\text{R}}_{x}\left(\text{control}\right)-\text{SG}{\text{R}}_{x}\left(\text{group}\right)}{\text{SG}{\text{R}}_{x}\left(\text{control}\right)}\cdot 100, \end{array}$$

where *I*_*x*_ = inhibition of specific growth in selected experimental group after *x* days of exposure, SGR_*x*_  (control) = mean specific growth rate in the control group, and SGR_*x*_  (group) = mean specific growth rate in selected experimental group.

Fulton's weight condition factor was calculated for each experimental group at every sampling time:

(3) $$\begin{array}{c} \text{FWC}=\frac{W\cdot 10^{5}}{{\text{TL}}^{3}}, \end{array}$$

where FWC = Fulton's weight condition factor, *W* = mass in selected experimental group (g), and TL = total length in selected experimental group (mm).

### 2.6. Samples Early Life Stage of Carp and Preparation of Postmitochondrial Supernatant

Toxicity tests on terbuthylazine-2-hydroxy were ended after 35 days. At the end of the tests fish were weighed and their length was determined. Samples were immediately frozen and stored at −80°C for analysis. Frozen tissue samples were weighed and homogenized (1 : 10, w/v) with an Ultra Turrax homogenizer (Ika, Germany) using 50 mmol/L potassium phosphate buffer, pH 7.0, containing 0.5 mmol/L EDTA. The homogenate was centrifuged at 4°C to obtain the postmitochondrial supernatant for antioxidant parameter analyses.

Total superoxide dismutase (SOD; EC 1.15.1.1) activity was determined spectrophotometrically by the method of S. Marklund and G. Marklund [15]. The catalase (CAT; EC 1.11.1.6) activity assay, using the spectrophotometric measurement of H_2_O_2_ breakdown at 240 nm, was performed following the method of Beers and Sizer [16]. Glutathione reductase (GR) activity was determined spectrophotometrically, measuring NADPH oxidation at 340 nm [17]. One unit of CAT or GR activity is defined as the amount of the enzyme that consumes 1 mol/L of substrate or generates 1 mol/L of product per min.

Protein levels were estimated spectrophotometrically by the method of Bradford [18] using bovine serum albumin as a standard.

### 2.7. Histopathology

Histopathology was evaluated in all experimental groups at the end of the experiment (day 35). Six whole fish from each group were placed in 10% buffered formalin, prepared with standard histological techniques, stained with hematoxylin and eosin, examined by light microscopy, and photographed using a digital camera.

### 2.8. Statistical Analysis

The statistical software program Statistica (ver. 8.0 for Windows, StatSoft) was used to compare differences among the test groups. Prior to analysis, all measured variables were checked for normality (Kolmogorov-Smirnov test) and homoscedasticity of variance (Bartlett's test). If those conditions were satisfied, a one-way ANOVA was employed to determine whether there were significant differences in measured variables among experimental groups. When a difference was detected (*P* < 0.05), Dunnett's multiple-range test was applied. If the conditions for ANOVA were not satisfied, a nonparametric test (Kruskal-Wallis) was used. The differences in accumulated mortality among the test groups were checked by using contingency tables (*χ* ^2^).

### 2.9. Evaluation of 35-Day LC50, LOEC, and NOEC

For the evaluation of 35-day LC50 values, a probit analysis was used based on mortality at different terbuthylazine-2-hydroxy concentrations. For the evaluation of LOEC and NOEC values, the probit analysis was based on inhibition of growth at different terbuthylazine-2-hydroxy concentrations; 35-day IC5 and 35-day IC10 values were used to express the NOEC and LOEC values, respectively. For evaluation, the EKOTOX 5.1 software (Ingeo, Liberec) was used.

## 3. Results and Discussion 

### 3.1. Hatching

Studies have reported that hatching can be affected by exposure to chemicals [19, 20]. In present study hatching began 4 days after the onset of exposure. The majority of eggs in all treatment groups hatched by day 6. No significantly negative effects of terbuthylazine-2-hydroxy at the concentrations tested (0.0029–3.5 mg/L) on hatching and embryo viability were observed. However, a marked influence was found on survival of larvae. Our results are in accord with results of Velisek et al. [10, 11], who found no change in carp hatching following exposure to triazine pesticides terbutryn and simazine.

### 3.2. Accumulated Mortality

**Figure 1:**
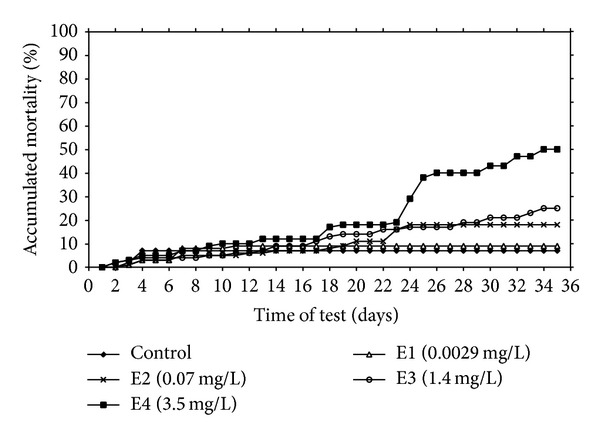
Accumulated percent mortality of common carp embryos, larvae, and juveniles after terbuthylazine-2-hydroxy exposure.

Significant (*P* < 0.01) differences in total accumulated mortality were found in fish exposed to the highest terbuthylazine-2-hydroxy concentration (3.5 mg/L), compared with controls (Figure 1). Massive mortality in this group occurred on days 23 and 26. Based on accumulated mortality in the experimental groups, values of lethal concentrations were estimated at 35-day LC50 = 10.9 mg/L terbuthylazine-2-hydroxy for early life stages of common carp. Most control larvae survived to 23 days after hatching, at which time it is likely that all yolk sac nutrients were exhausted. This is in accordance with data concerning the so called “point of no return”—the moment when the larvae irreversibly lose ability to feed and die even if provided with food. In common carp larvae the point of no return occurs about day 15 after hatching [21], and starvation-induced mortality occurs after that time.

### 3.3. Length and Weight Growth Parameters

**Figure 2:**
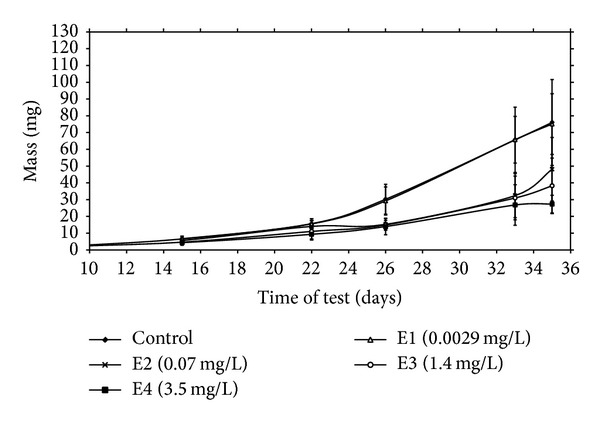
Mean mass ± SD of common carp larvae (juveniles) after terbuthylazine-2-hydroxy exposure.

**Figure 3:**
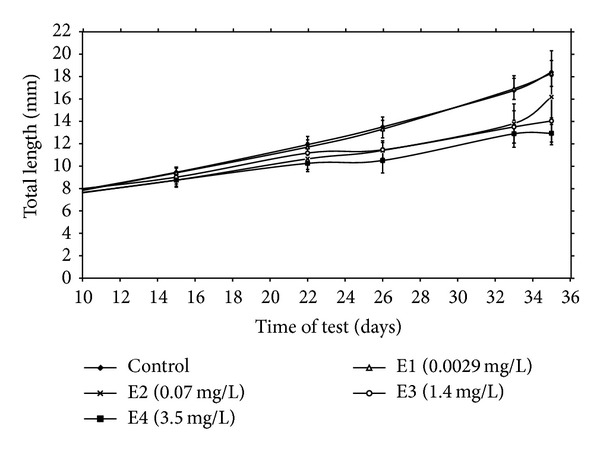
Total length ± SD of common carp larvae (juveniles) after terbuthylazine-2-hydroxy exposure.

Growth can be considered a more sensitive measure than mortality. Growth represents an integration of a variety of physiological and environmental factors. It provides a sensitive gauge of environmental conditions and is important for reviewing the success with which organisms adapt to their environment [22]. Mass and total length of fish as related to terbuthylazine-2-hydroxy concentration in water are shown in Figures 2 and 3. In the group 4 fish beginning on day 15 of exposure showed significantly (*P* < 0.01) lower total length compared with controls. Beginning on day 26 of exposure, in groups 2, 3, and 4 fish, terbuthylazine-2-hydroxy caused significantly (*P* < 0.01) lower mass and total length compared with controls. Specific growth rates and inhibition of growth are shown in Table 1. Inhibition of growth in the group exposed to the two highest tested concentrations (1.4 and 3.5 mg/L) was 17.66% and 28.32%, respectively, compared to control. Based on inhibition of growth in the experimental groups, lowest observed effect concentration (LOEC) = 0.002 mg/L terbuthylazine-2-hydroxy and no observed effect concentration (NOEC) = 0.0001 mg/L terbuthylazine-2-hydroxy. Fulton's condition factors of common carp exposed to terbuthylazine-2-hydroxy showed no differences from untreated fish. This is supported by another study that showed a decreased growth after terbutryn and simazine exposure in carp [10, 11]. Growth reductions after terbuthylazine-2-hydroxy exposure might delay maturation and reproduction as well as increase the susceptibility of young fish to predation and disease. Their ability to obtain food and to compete for suitable habitats might also be reduced.

### 3.4. Early Ontogeny

The developing fish embryo and early larval stages have been shown to be especially sensitive indicators of many types of aquatic pollution. A chemically induced adverse effect on embryonic stages is based on developmental events, for example, organogenesis [23, 24]. Although we have diverse information on the toxicity of terbutryn in adult stages of fish, little is known of effects of this compound on embryonic development in early life stages of common carp at environmental concentrations. The developmental stages observed at the sampling times in all tested concentrations and controls are listed in Table 2. Significant differences in early ontogeny among test groups were observed for the duration of the trial. Fish from two highest tested concentrations (1.4 and 3.5 mg/L) were delayed in development compared with the control group. The percent of individuals in larval stages (L4b or L5) increased with higher concentrations of terbuthylazine-2-hydroxy, whereas the majority of control fish reached the juvenile stage. Our results are in accord with results of Velisek et al. [10, 11], who found differences in early ontogeny of carp following exposure to triazine pesticides terbutryn and simazine.

### 3.5. Macroscopic Morphological Anomalies

In fish, developmental malformations have been linked to the presence of environmental pollutants such as persistent organochlorines and pesticides [25]. In the present study similar morphological anomalies were found in fish in both experimental and control groups. These included axial and/or lateral curvature of the spine (lordosis and scoliosis), yolk sac deformity, and body shortening. The incidence of these anomalies was 0.2%, which could be considered a spontaneous appearance. In other studies, pesticide effects that have been observed on fish embryonic development have included malformations in myoskeletal development (such as notochord abnormalities of degeneration), defects along the rostral-caudal body axis, curvature of the vertebral column, and edemas in the pericardial area or yolk sac [26–28].

### 3.6. Antioxidant Response

Numerous studies have demonstrated that exposure to triazine herbicides affects the antioxidant defense system in fish, causing an imbalance between reactive oxidative system production and elimination and resulting in oxidative stress and organism damage [9, 29, 30]. The first line of defense against oxidative stress consists of the antioxidant enzymes SOD, CAT, and GPx, which convert superoxide anions (O_2_^−^) into H_2_O_2_ and then into H_2_O and O_2_ [31]. Effect of chronic exposure to terbuthylazine-2-hydroxy on antioxidants responses SOD, CAT, and GR in homogenate on early life stages of carp are in Table 3. Significant differences from the control value (*P* < 0.01) were seen in Groups E1, E2, E3, and E4 on homogenate early life stages of carp in SOD activity. SOD activity was significantly lower in all groups tested terbuthylazine-2-hydroxy compared with the control group. In CAT and GR activities changes were not observed in tested groups. Superoxide dismutase is an antioxidant enzyme important in inhibiting oxyradical formation and is used as a biomarker to indicate oxidative stress [32, 33]. In our test, decrease in SOD activity may be due to increased generation of ROS induced by terbuthylazine-2-hydroxy exposure. Similar changed activities of SOD in carp tissues after pesticides exposure have also been reported by other authors: Oruc et al. [34], Oruç and Usta [35], Velisek et al. [36], and Stara et al. [29, 30].

### 3.7. Histopathology

Generally, triazine pesticides have a direct effect on kidney structure and function in freshwater fish [10, 11, 36–38]. No histopathological changes were demonstrated in gills and liver following exposure to terbuthylazine-2-hydroxy. The majority of histological changes were observed in caudal kidney in groups E3 (1.4 mg/L) and E4 (3.5 mg/L) compared to control fish. Fish exposed to highest tested levels of terbuthylazine-2-hydroxy showed alteration of tubular system that included destruction of tubular epithelium with casts, vacuolization of tubular epithelia, and disintegration of glomeruli. The kidney is important for the maintenance of a stable internal environment with respect to water and sodium chloride, for excretion, and, partially, for the metabolism of xenobiotic [39]. It is evident that renal alteration was related to terbuthylazine-2-hydroxy exposition, while liver and gill were not affected. On the basis of our findings it is possible to describe terbuthylazine-2-hydroxy as a primary nephrotoxic substance.

## 4. Conclusions

Chronic terbuthylazine-2-hydroxy exposure of early-life stages of common carp affected their growth rate, early ontogeny, antioxidant enzyme, and histology. Some of the changes (early ontogeny, histology) were observed only at two higher exposures (1.4 and 3.5 mg/L), but changes founded in growth rate and antioxidant enzyme were affected in fish exposed to the lowest concentration tested (i.e., 0.0029 mg/L), which is that reported in Czech rivers in recent years. Aquatic environment may be polluted by many substances, the effects of which can be potentiated with combined exposures. For detailed elucidation of terbuthylazine-2-hydroxy effects further research is necessary. This research should be focused not only on the studies of effects of terbuthylazine-2-hydroxy alone but also in view of possible synergic or potentiation effect.

## Figures and Tables

**Table 1:** Growth rate and fish mortality results of the 35-day embryo-larva toxicity test on common carp after terbuthylazine-2-hydroxy exposure.

	Fish group				
	Control	E1	E2	E3	E4
Terbuthylazine-2-hydroxy (mg/L)	—	0.0029	0.07	1.4	3.5
*m*_9_ (mean ± SD, mg)	2.32 ± 0.46	2.34 ± 0.54	2.08 ± 0.43	2.11 ± 0.60	2.27 ± 0.33
*m*_35_ (mean ± SD, mg)	76.03 ± 25.58	75.08 ± 18.12	48.11 ± 19.93*	38.23 ± 16.55*	27.34 ± 5.28*
SGR	13.31	13.35	11.88	10.96	9.54
*I* (%)	—	−0.30	10.74	17.66	28.32
Total mortality (%)	7	9	18	25	50

*m*_9_, *m*_35_: mean fish mass in selected group after 9 and 35 days exposure; SGR: mean specific growth rate in selected group after 26 days exposure; *I*: inhibition of specific growth in selected group after 26 days exposure; SD: standard deviation. *Experimental groups significantly (*P* < 0.01) different from the control group.

**Table 2:** Developmental periods (DPS) during the 35-day embryo-larva toxicity test on common carp.

	Fish group				
	Control	E1	E2	E3	E4
Terbuthylazine-2-hydroxy (mg/L)	—	0.0029	0.07	1.4	3.5
Times (day)	DPS	DPS	DPS	DPS	DPS
9	Ec9-L1	Ec9-L1	Ec9-L1	Ec9-L1	Ec9-L1
15	L2-L3b	L2-L3b	L2-L3b	L2-L3a	L1-L2
22	L4a-L5	L4a-L5	L4a-L5	L4a-L4b	L3-L4a
26	L5-L6	L5-L6	L5-L6	L4a-L4b	L4a-L4b
33	L6-J1	L6-J1	L6-J1	L5-L6	L4b-L5
35	J1	J1	J1	L5-L6	L4b-L5

**Table 3:** Effect of terbuthylazine-2-hydroxy exposure on superoxide dismutase (SOD, nmol NBT/min/mg protein), catalase (CAT, µmol H_2_O_2_/min/mg protein) and glutathione reductase (GR, nmol NADPH/min/mg protein), activity in homogenate of early life stages of carp.

	Fish group				
	Control	E1	E2	E3	E4
Terbuthylazine-2-hydroxy (mg/L)	—	0.0029	0.07	1.4	3.5
SOD	0.3819 ± 0.1073	0.1726 ± 0.0268*	0.1880 ± 0.0447*	0.1182 ± 0.0626*	0.0434 ± 0.0136*
CAT	0.1254 ± 0.0826	0.1296 ± 0.0678	0.1477 ± 0.0641	0.1640 ± 0.0512	0.1825 ± 0.0696
GR	0.1968 ± 0.1726	0.1823 ± 0.1346	0.1037 ± 0.0998	0.1789 ± 0.1276	0.1932 ± 0.1470

*Experimental groups significantly (*P* < 0.01) different from the control group.
